# Does government institutional reform deter corporate tax evasion? Evidence from China

**DOI:** 10.1371/journal.pone.0273372

**Published:** 2022-12-08

**Authors:** Junbing Xu, Minling Zhu, Shengying Song, Yunxi Wu

**Affiliations:** 1 NewHuadu Business School, Minjiang University, Fuzhou City, Fujian Province, China; 2 Economic College, Hunan Agricultural University, Changsha City, Hunan Province, China; 3 Institute of Finance and Economixs, Jimei University, Xiamen City, Fujian Province, China; 4 School of Economics and Management, Fuzhou University, Fuzhou City, Fujian Province, China; Universiti Malaysia Sabah, MALAYSIA

## Abstract

Exploiting the quasi-natural experiment of the social insurance collection system reform implemented in China in 2000, based on data from China’s industrial enterprise database from 1998 to 2006, we use the difference-in-differences method to test the impact of changing the social insurance collection institution on corporate tax evasion. We find that changing the social insurance collection institution from the social security department to the local tax department significantly deters corporate tax evasion. A series of robustness tests also support this conclusion. The reason is changing the social insurance collection institution to the local tax department increases its’ social insurance information of the enterprise, and reduces the information asymmetry between the enterprise and the collection institution. Furthermore, we find that the impact of changing the social insurance collection institution on corporate tax evasion is more evident in the samples of labor-intensive enterprises, low labor cost enterprises, and enterprises under the jurisdiction of the local tax department. These results indicate that government institutional reform is a valid way to reduce the information asymmetry between the government and enterprises, which will finally deter corporate tax evasion.

## 1. Introduction

Taxation is the most important form of government fiscal revenue, and corporate tax evasion will inevitably reduce it. Therefore, it is essential to study “why corporate avoid” and provide regulation advice to policymakers. Existing literature discusses the causes of corporate tax evasion on both internal and external factors of firm. Earlier research tended to focus on firm internal factors that may result in tax evasion [1]. With the development of tax evasion research, the research frontier in this field is shifting from the study of firm internal factor to the external factors. Kleven et al. (2011) is the seminal work that causally identify the impact of external factor on corporate tax evasion behavior [2]. They find that the threats of audit had a large effect on tax compliance, and the third-party reporting environment also largely improve tax compliance. Since then, researchers have discussed the external factors of corporate evasion from the perspectives of the probability of audit [3, 4], enforcement intensity [5–7], regulatory informatization [8–10], public disclosure [11, 12], and deterrence [13–19]. In fact, these studies only measure the external supervision of corporate tax evasion from different perspectives. The intensity of external supervision is directly related to a country’s enforcement capacity and enforcement costs. However, for low- and middle-income countries, the ability to increase the intensity of tax external supervision is limited [20]. Therefore, the policy advices that these studies can provide to low- and middle-income countries are limited. Institution is an important factor affecting corporate behavior. As developing country, China has experienced a 20-year battle over social insurance collection institutions setting, from 1999 to 2019. This provides an excellent quasi-natural experiment for us to study the impact of government institution settings on micro-enterprises. As far as we known, no studies have discussed the impact of government institution settings on corporate tax evasion.

China’s social insurance system began in 1986 with the “Notice of the State Council on Issuing Four Regulations for Reforming the Labor System” issued by the State Council of China. After that, China’s social insurance premiums are uniformly collected by the social security department. In 2000, due to the difficulty in collecting social insurance premiums, China officially implemented a reform of the social insurance collection institution. The reform in 2000 declare that social insurance premiums can be collected either by tax authorities or social security department, which all depends on the decision of provincial government. Therefore, after the social insurance collection reform in 2000 and before China officially implemented the unified collection of social insurance premiums by the tax authorities in 2019, the collection institutions of social insurance premiums in different provinces in China might be different. We use this natural experiment to study how changing the social insurance collection institution affects corporate tax evasion.

In theory, changing the social insurance collection institution from the social security department to the local tax department has two possible impacts on corporate tax evasion. First, changing the social insurance collection institution will increase the verification rate of enterprise social insurance, which may enhance social insurance premium collection by government departments. This reduces the free cash flow of enterprises, thus causing financial pressure. To “neutralize” this financial pressure, enterprises may increase tax evasion [21]. Second, after the social insurance collection institution is changed to the local tax department, the latter will obtain more social insurance information of the enterprise [22], which will help the tax department to estimate the actual employment situation and labor cost by using enterprise social insurance data. The direct consequence is that the behavior of enterprises using labor costs to avoid tax is more likely to be detected by the tax department. Meanwhile, after the tax department discovers the taxation problem of the enterprise through social insurance data, it will continue to conduct an audit on the overall business status and accounting transactions of the enterprise. At this time, other tax evasion methods adopted by enterprises are also easier to detect.

Empirically, how will changing the social insurance collection institution affect corporate tax evasion? With the help of the reform of the social insurance collection institution officially implemented in China in 2000, based on the data of China’s industrial enterprise database from 1998 to 2006, using the difference-in-differences (DID) method, we examine the impact of changing the social insurance collection institution on corporate tax evasion. Based on the empirical research of this natural experiment, we find that: (1) Changing the social insurance collection institution from the social security department to the local tax department reduces the tax evasion of Chinese enterprises. This conclusion remains robust even after a series of robustness tests. (2) In the mechanism analysis, we find that after the social insurance collection institution was changed to the local tax department, the external governance of the enterprise was strengthened. This reduces the information asymmetry between the tax department and the enterprise, and thus deters corporate tax evasion. (3) Furthermore, we find that the impact of changing the social insurance collection institution on corporate tax evasion is more evident in local tax companies, labor-intensive companies, and low labor cost companies.

This study makes three main contributions to the literature. First, we study the factors affecting corporate tax evasion behavior from a new perspective. The literature mainly examines the influencing factors of corporate tax evasion from a corporate governance perspective, including corporate size [23–26], market power [27], the nature of corporate property rights [28], reputation [29], executives’ military or political experience [30], and so on, or from a macro-level perspective, such as the uncertainty of the external political environment [31], financial systems [32], financing environment [33], and minimum wage law [34]. As for the external supervision factors, existing literature mainly study from the perspective of the probability of audit [3, 4], enforcement intensity [5–7], regulatory informatization [8–10], public disclosure [11, 12], and deterrence [13–19]. We proof that how changing the social insurance collection institution affects corporate tax evasion, providing a novel perspective for a deep understanding of the intricate driving factors behind corporate tax evasion.

Second, this study enriches the literature on the impact of changing the social insurance collection institution on micro-enterprises. Studies on changing the social security collection institution have mostly discussed its impact on local social security funds, or labor-capital ratio [35] and the impact on the entry and exit rates of enterprises [36]. To the best of our knowledge, no study has focused on the impact of changing the social insurance collection institution on corporate tax evasion until now. Our study enriches research on the impact of government institutional reform on micro-enterprises.

Third, this study provides valid policy implications for the low- and middle-income countries to deter corporate evasion. The existing literature about the external supervision factors of tax evasion mainly study from the perspective of the supervision strength [10], but improving the supervision factors is hard to implement for low- and middle-income countries. The literature review of Mascagni (2018) also indicate that this research field requires more evidence from low- and middle-income countries [20]. Our study uses the data from China and provide a possible policy basis for corporate tax evasion from the perspective of valid social insurance collection institutions setting.

The remainder of this paper proceeds as follows: Section 2 presents the literature review and research hypotheses; Section 3 briefly reviews the change process of China’s social insurance collection institution; Section 4 includes the data and methodology; Section 5 presents the estimation results and corresponding discussions; and finally, Section 6 provides the conclusions and implications.

## 2. Literature review and research hypothesis

### 2.1. Literature review

Corporate governance is one of the factors that influence corporate tax evasion. For enterprises, tax evasion has the effect of increasing cash resources; therefore, enterprises are motivated to evade tax. However, Desai and Dharmapala (2007) and Desai et al. (2007) report that creating complex transactions not only help enterprises to evade tax but also aggravate the information asymmetry between management and shareholders [5, 37]. This enables the enterprise management to seek individual benefits. This hidden danger increases shareholders’ supervision costs. If executive compensation makes the interests of managers and shareholders consistent, and if shareholders tend to avoid tax to increase the value of the company, the theory predicts that there is a positive correlation between incentive compensation and tax evasion [38–40]. Phillips (2003) conducted a more in-depth study on this issue from the perspective of managers’ roles. He found that incentive compensation plans based on CEO after tax performance could provide incentives for companies to evade tax [41]. Consistent with Phillip’s (2003) research results, Armstrong et al. (2012) also did not observe a significant relationship between general CEO compensation and corporate tax evasion [42]. In other words, corporate tax evasion is related to whether CEO compensation is linked to their performance [43, 44]. In addition, the existing literature discusses the corporate governance factors that influence corporate tax evasion from the proportion of external directors [45], dual ownership structure [28], and proportion and concentration of family ownership [46, 47].

In addition to the internal governance structure of enterprises, many other factors, such as enterprise scale and market power, also affect corporate tax evasion behavior. Kubick et al. (2015) find that enterprises with market power are more likely to evade taxes because they have comparative advantages in the market and can better understand the extent to which tax evasion strategies are beneficial [27]. Additionally, larger companies may engage in more tax evasion activities [24]. Although large companies may face more scrutiny, they may also have resources and greater incentives to evade taxes [25, 26]. Whether an enterprise conducts transnational operations may also affect its tax evasion behavior. Multinational enterprises can transfer taxable income to jurisdictions with lower taxes because multinationals have more opportunities to evade taxes through structured transactions [11, 24, 48]. Reputation is another important factor that affects corporate tax evasion. Business owners may be tempted to reduce tax evasion, as tax evasion, if detected, can negatively impact a business’s reputation [29]. Higgins et al. (2015) find that a company’s business strategy is associated with tax evasion. They found that risk-averse (robust) firms were more (less) likely to evade taxes [49]. External financial constraints, external financial resources [50], and political connections [51–55] are also important external factors that influence corporate tax evasion.

The research most relevant to this study focuses on the external supervision of corporate tax evasion. Studies have found that external supervision reduces corporate tax evasion [47, 56]. Desai et al. (2007) found that Russian companies increased their taxes and decreased related party transactions after Putin became the president because his inauguration has made Russia’s tax enforcement stricter [5]. Hoopes et al. (2012) found that when the Internal Revenue Service (IRS) was more regulated, U.S. public companies had less tax planning. External investors can also play an important role in supervising enterprises’ tax behaviors [7]. Khurana and Moser (2013) found that external investors with expertise and experience could monitor managers’ rent-seeking behavior, thereby reducing corporate tax evasion [57]. Tian et al. (2016) and Kanagaretnam et al. (2018) also find that the media has a supervisory effect on corporate tax behavior [58, 59]. Media exposure can impose reputational costs on companies; therefore, companies that are subject to greater media scrutiny have lower levels of tax evasion. Besides these, deterrence is the most effective way to increase tax compliance in the short run [2, 13]. However, the effectiveness of deterrence largely depends on whether deterrence can be achieved. For example, Ortega and Scartascini (2016) find that the method may affect the effectiveness of deterrence [42]. According to their study, taxpayers may update their perceived probability of detection depending on the delivery method chosen by the authority, so that more selective and more costly ones may have a higher deterrence effect. Unfortunately, for low- and middle-income countries, increasing costs to enhance the effectiveness reduces the viability of deterrence.

As mentioned, studies have investigated internal or external factors that influence corporate tax evasion, and also investigated the links between changing the social insurance collection institution and the income of social security funds or corporate social security contributions, but they have overlooked the impact of this change on corporate tax evasion. Studies on the factors influencing corporate tax evasion have also neglected the influence of government institution setting. In fact, the setting of external institutions is an important factor for enterprise taxation supervision, which is directly related to the tax planning behavior within the enterprise. Changing the social insurance collection institution could reduce the information asymmetry between tax authorities and enterprises, which may finally affect corporate tax evasion behaviors. Accordingly, we study how changing the social insurance collection institution affects corporate tax evasion via information channels based on China’s industrial enterprise database.

### 2.2. Research hypothesis

In this section, we analyze how changing the social insurance collection institution affects corporate tax evasion through information channels.

#### 2.2.1. External supervision and corporate tax evasion

Hope et al. (2013) study the relationship between corporate information disclosure and corporate tax evasion, finding that companies that chose to stop disclosing geographic earnings in their financial reports had lower effective tax rates globally [11]. However, upon using the Schedule M-3 rule implemented in 2004 as an exogenous shock, they found that the relationship between tax evasion and non-disclosure decreased after Schedule M-3’s implementation. This is because the Schedule M-3 rule requires businesses to conduct a detailed reconciliation of book income against tax income, thereby complicating tax evasion. Hanlon et al. (2014) studied the relationship between tax enforcement and financial reporting quality, finding that the tax enforcement intensity was positively correlated with the financial reporting quality [60]. This conclusion is consistent with Desai et al.’s (2007) and Atwood et al.’s (2012) predictions [5, 6]. Hoopes et al. (2012) found that IRS audits significantly reduced tax evasion, a view also supported by Chen and Gavious (2015) [7, 61]. This shows that the regulatory means and intensity of state institutions impact corporate tax evasion. In addition, factors such as public scrutiny [12], external company audits [4], periodic reviews by the U.S. Securities and Exchange Commission [19] may reduce corporate tax evasion. Therefore, strengthening external supervision can prevent corporate tax evasion.

#### 2.2.2. Information asymmetry and corporate tax evasion

Kubick et al. (2017) found that the closer a company’s headquarters was to the IRS regional manager’s office, the more tax evasion the company could reach. They explained that firms closer to the IRS offices had better access to information and thus could better prepare for IRS audit oversight. Numerous studies have shown that people can gain valuable information from communities wherein they conduct their daily activities [62]. Paterson and Meegan (2019) also found that when supervisors (e.g., analysts, investors, auditors, and regulators) were close to the firms they inspected, their audit results were better [63]. Chen et al. (2021) studied the impact of the mandatory use of extensible Business Reporting Language (XBRL) for financial reporting to reduce the information processing cost of the IRS on corporate tax evasion, finding that the adoption of XBRL in financial reporting can significantly reduce tax evasion [64]. The aforementioned research shows that information plays an important role in corporate tax evasion. When information asymmetry intensifies, corporate tax evasion may increase.

Based on the foregoing analysis, we propose the following two hypotheses:

**Hypothesis 1. Changing the social insurance collection institution from the social security department to the local tax department will reduce corporate tax evasion**.
**Hypothesis 2. Changing the social insurance collection institution will reduce corporate tax evasion by reducing the information asymmetry between the collection institution and enterprises**.

## 3. Institutional background

China’s social insurance collection system has gone through a series of reforms from its establishment, improvement and finalization. It is precisely because of this series of changes in the social insurance collection system that we have an opportunity to study the impact of government institution settings on corporate tax evasion today. In general, the improvement of China’s social insurance collection and payment system has gone through four stages.

The first stage is the stage of “system establishment—notification of regulations” of social insurance collection, where the country is in the ascendant stage, having just begun to explore the establishment of a social security system. After the founding of the People’s Republic of China, although China’s social security undertakings had developed to a certain extent, the overall level was relatively low. It was not until July 1986 that China’s State Council issued the “Notice of the State Council on Issuing Four Provisions for Reforming the Labor System” that the corresponding regulations were officially adopted to build China’s social security system. The notice requires state and public institutions to pay endowment and business insurance to employees when hiring workers. Therefore, the promulgation of this notice also marks the formal establishment of China’s social security system, which is of epochal significance.

The second stage is the “provincial pilot—interim regulation” stage of social security collection, where various localities have conducted many useful experiments to develop corresponding systems. Article 6 of the “Interim Regulations on the Collection and Payment of Social Insurance Premiums” promulgated by the State Council in 1999 stipulates that the collection institution of social insurance premiums shall be prescribed by the provincial people’s government, which may be collected by tax authorities or by the social insurance department. At this stage, although some regions tried to ensure that pensions were paid in full and on time, the pressure was too great, and the state’s supporting financial funds could not meet the needs. Therefore, some regions have conducted useful explorations and experiments through the taxation department to collect social insurance premiums.

The third stage is the “Uniform Collection-Social Insurance Law” stage of social security collection, where the state clarified the legal form of implementing the unified collection of social security. Enacted in 2010, Article 59 of the Social Insurance Law stipulates that social insurance premiums shall be collected uniformly, and the implementation steps and specific methods shall be prescribed by the State Council. However, the law does not clearly stipulate whether social security should be collected by the taxation or social security department.

The last stage is the “tax collection-reform plan” stage of social security collection, where China clarifies that the collection of social security is levied by the tax authorities. In March 2018, the central government issued the “Plan for Deepening the Reform of Party and State Institutions.” The plan proposed to pay attention to the improvement of the efficiency of collection and management of social insurance funds and to hand over the expenses related to social insurance to a unified institution for management. In other words, the tax department should be the main social security collection department. Accordingly, China officially launched an exploration of the reform of the social security collection institution. On July 20, 2018, the “National Tax and Local Tax Collection and Administration System Reform Plan” was successively promulgated. The plan clearly stipulated that from January 1, 2019, a system of uniform collection of social insurance by the tax department should be officially implemented. Thus far, the decades-long battle over the subject of social security collection has ended with a unified collection by the tax department.

As mentioned, Article 6 of the “Interim Regulations on the Collection and Payment of Social Insurance Premiums” promulgated by the State Council in 1999 stipulates that the collection institution of social insurance premiums shall be determined by the provincial people’s government. Social insurance premiums can be collected either by tax authorities or social insurance institutions. Therefore, after the promulgation of the aforesaid interim regulations and before China officially implemented the unified collection of social insurance premiums by the tax authorities in 2019, the collection institutions of social insurance premiums in different provinces in China might be different. We use this natural experiment to study how changing the social insurance collection institution affects corporate tax evasion.

## 4. Research design

### 4.1. Samples and data sources

We use the data of the Chinese Industrial Enterprises Database from 1998 to 2006. The China Industrial Enterprise Database is the original data at the enterprise level, and the statistical objects are large and medium-sized manufacturing enterprises with an annual turnover of 5 million yuan and above. Industrial statistical indicators include major technical and economic indicators such as industrial added value, industrial output value, and industrial sales output value. Enterprise financial indicators include income tax payable, value-added tax payable this year, paid-in capital, operating profit, total profit, total owner’s equity, product sales revenue, product sales cost, product sales tax and surcharges, etc. The database is collected from the National Bureau of Statistics.

Based on the Chinese Industrial Enterprises Database, we perform the corresponding processing of the data at the enterprise level. Specifically, according to the legal person code, enterprise name, address, telephone number, and other information of the investigated enterprises, we identified and matched the enterprises in different years, and constructed unbalanced panel data of the Chinese Industrial Enterprise Database from 1998 to 2006. Second, we perform basic data cleaning, such as deleting observations that lack key variables, eliminating observations that are not logical in financial indicators (total assets are less than the net value of the company’s fixed assets, total assets are less than the current assets of the company, and the original value of fixed assets is less than 1 million, the number of employees is less than 8, enterprises established before 1949, etc.), deleting the samples whose actual tax rate is less than 0 and greater than 1, and deleting the samples whose enterprises have only one year of observation. Finally, we obtain 5,909,117 observations in the range of 1998–2006.

### 4.2. Empirical model and variables

Drawing on previous studies [34], the empirical model of this study is designed as follows.


$${ERT}_{p,i,t}=\alpha_{0}+\beta \cdot {So\_In}_{p,t}+\gamma {\cdot Control}_{p,i,t}+\delta_{t}+\theta_{i}+\varepsilon_{p,i,t}$$ (1)

In Model (1), the subscript *p* represents province, *i* represents enterprise, and *t* represents year. *ERT* is the dependent variable, and *So_In* is the core explanatory variable. *δ*_*t*_ is year fixed effect and *θ*_*i*_ is corporate fixed effect. We cluster the standard error to enterprise level in the benchmark model.

#### 4.2.1. Dependent variable

*ERT* is the dependent variable, actual tax rate, which is used to measure enterprise tax evasion. Referring to Liu and Wu (2014) and Porcano (1986) [65, 66], we use the ratio of the income tax payable to pre-tax accounting profit as a proxy indicator to measure *ERT*.

#### 4.2.2. Core explanatory variable

*So_In* is the core explanatory variable in the benchmark model, which represents changing the social insurance collection institution. Specifically, when the social insurance institution in year *t* in province *p* is changed to the tax department, the value is 1; the value of this province in other years after the change is also set to 1, and 0 in other years. This is a two-dimensional variable that acts as an interaction term in the DID method. Therefore, *β* is our coefficient of focus. If changing the social insurance collection institution to the tax department can significantly reduce corporate tax evasion, it is expected that *β* will be significantly positive.

#### 4.2.3. Control variables

Control is a series of control variables. Referring to research on corporate tax burden, we select the following control variables. The control variables at the provincial level: For per capita gross domestic product (GDP) growth rate (*Gropgdp*), Li et al. (2016) believe that the regional economic situation will lead the local government to adopt corresponding tax policies to intervene in the tax burden of enterprises, as well as population size (*Popu*), which is measured by the natural logarithm of the population in 10,000 units [67]. Wilson (1991) argues that the competitive equilibrium tax burden is higher in regions with larger populations. The control variables at the enterprise level are the nature of property rights (*Soe*), 1 for state-owned and 0 for non-state-owned [68]. Wu et al. (2012) believe that the nature of corporate property rights makes enterprises show the effects of “tax competition” and “tax grabbing,” which may affect the tax burden of enterprises [69]. For company size (*Size*), we use the natural logarithm of the company’s total assets to measure. Zimmerman (1983) argues that large companies are vulnerable to attention, leading to higher effective tax rates [23]. For financial leverage (*Lev*), we use total liabilities divided by total assets to measure. For profitability (*Roa*), we use net profit divided by total assets to measure. Spooner (1986) reports that profitability has an important impact on corporate tax burden [70]. We use the fixed assets ratio (*NetFi*), which is measured by dividing net fixed assets by total assets, and the inventory ratio (*Inve*), which is measured by dividing net inventory by total assets. Gupta and Newberry (1997) argue that the depreciation and treatment of assets and inventories will affect the corporate tax burden [71]; company age (*Age*), we use the natural logarithm of the company’s establishment to date. The size of the company’s employment (*Employ*) is measured by the natural logarithm of the number of employees in the company. Meanwhile, to control the self-selection problem of the treatment group, that is, which characteristics will cause some provinces to choose to change the social insurance collection institution, we add the interactions of social security payment pressure in 1997 (social security fund income-social security fund expenditure), per capita GDP (per capita GDP), and the industrial share (industrial output/GDP) with the time trend as a control variable. In addition, we add a year fixed effect (*δ*_*t*_) and corporate fixed effect (*θ*_*i*_) in the model.

### 4.3. Summary statistics

Table 1 reports the descriptive statistics of the main variables. The mean and standard deviation of the explained variable *ERT* are 0.265 and 0.156, respectively, which are close to the actual tax rate index value in most studies. From the standard deviation of other control variables, a certain degree of difference in the characteristic control variables between provinces and enterprises is probable, and the actual tax burden of enterprises may be affected by this difference.

**Table 1:** Descriptive statistics of the core variables.

Variable	Sample	Mean	Standard Error	Min	Max
Panel A Provincial variable					
*Gropgdp*	222	0.016	0.050	-0.095	0.314
*Popu*	222	17.14	0.953	14.74	18.39
Panel B Enterprise variables					
*ERT*	590911	0.265	0.156	0	1
*Size*	590911	9.806	1.457	4.625	20.06
*Age*	590911	2.090	0.798	0	4.127
*Employ*	590911	4.905	1.145	2.079	12.58
*Inve*	590911	0.179	0.160	-1.027	4.885
*Roa*	590911	0.539	0.255	-1.344	31.16
*Lev*	590911	0.116	0.277	-5.770	37.97
*Soe*	590911	0.087	0.281	0	1
*NetFi*	590911	0.349	0.209	0	1

## 5. Empirical results and discussion

### 5.1. Benchmark regression

As we mentioned in the institutional background, China’s social insurance has gone through the process of being collected by the collection institutions designated by each province to the unified collection by the local tax department. In the process of appointing social insurance collection institutions by each province, many cities have handed over the social insurance previously collected by the social security department to the local taxation department. This provides an opportunity for us to use the DID approach to assess the impact of government institution settings on corporate tax evasion. To the best of our knowledge, no studies have discussed the impact of government institution settings on corporate tax evasion.

In the baseline regression part, taking China’s local social insurance collection institution reform from 2000 to 2019 as a quasi-natural experiment, using the DID method, we evaluate the impact of changing the social insurance collection institution from the social security department to the local tax department on corporate tax evasion. To reduce the impact of unobservable firm heterogeneity on the regression results, we controlled for firm-year fixed effects in the regression and used firm-level cluster standard errors. Table 2 reports the impact of changing the social insurance collection institution from the social security department to the local tax department on corporate tax evasion. Column (1) of Table 2 presents the univariate empirical result that controls only time and firm-level fixed effects; Column (2) shows the empirical result of adding the province control variables based on the model in Column (1); Column (3) reports the empirical result of adding enterprise control variables based on the model in Column (2); Column 4 adds the interaction term of the social security payment pressure in 1997, per capita GDP, and industry share with the time trend based on the model in Column (3) to control the deviation of the policy pre-variables. The core explanatory variable *So_In* has a significant positive correlation with the actual corporate tax *ERT* at the 1% statistical level in all models, and the coefficient value of the core explanatory variable changes a little in each column. Therefore, the estimation of the core explanatory variable *So_In* is relatively robust. This indicates that changing the social insurance collection institution from the social security department to the local tax department will significantly increase the actual tax rate of corporate income tax and deter the tax evasion behavior of an enterprise. This also shows that, in developing counties where government need to invest huge cost to improve governance capacity like China, compared with the social security department, the local tax department can increase the tax verification rate when collecting social insurance, thereby reducing corporate tax evasion.

**Table 2:** Benchmark regression: Social insurance collection institution and corporate tax evasion.

Explained variable	*ERT*			
Explanatory variables	(1)	(2)	(3)	(4)
*So_In*	0.006***	0.006***	0.005***	0.007***
	(4.702)	(4.592)	(3.732)	(5.045)
*Gropgdp*		0.004	0.003	0.001
		(0.812)	(0.558)	(0.246)
*Popu*		0.063***	0.033***	-0.033***
		(7.691)	(4.092)	(-3.464)
*Size*			-0.013***	-0.013***
			(-18.456)	(-18.368)
*Age*			0.001**	0.001
			(2.183)	(1.467)
*Employ*			0.003***	0.003***
			(4.924)	(4.930)
*Inve*			0.008***	0.009***
			(3.388)	(3.612)
*Roa*			0.022***	0.022***
			(10.480)	(10.448)
*Lev*			-0.041***	-0.041***
			(-11.474)	(-11.460)
*Soe*			0.011***	0.011***
			(5.181)	(4.861)
*NetFi*			0.003	0.003
			(1.210)	(1.359)
Constant	0.263***	-0.848***	-0.227	0.754***
	(623.259)	(-5.871)	(-1.558)	(4.599)
Firm FE	YES	YES	YES	YES
Year FE	YES	YES	YES	YES
Other control variables	NO	NO	NO	YES
Observations	590911	590911	590911	590911
Adj-R2	0.346	0.346	0.350	0.350

Note

(1) The T-statistic after clustering is in parentheses; (2) ***, **, and * indicate significance at the 1%, 5%, and 10% levels, respectively; (3) The social security payment pressure in 1997 is measured by social security fund income divided by social security fund expenditure, per capita GDP is measured by the logarithm of per capita GDP, and industry share is measured by industrial output/GDP.

### 5.2. Robustness check

To ensure the reliability of the benchmark regression conclusions, we perform the following robustness tests to verify the empirical results presented in Table 2, which includes the parallel trend test, bacon decomposition, PSM-DID test, Heckman two-step method and other robustness tests.

#### 5.2.1. Parallel trend test

One premise of using DID is that the corporate evasion indicator *ERT* (explained variable) should satisfy the parallel trend assumption. As changing the social insurance collection institution is a multipoint policy, we can only use the event analysis method to test the parallel trend of the DID approach. We use the year before the reform as a benchmark. The empirical results are presented in Table 3. All the coefficients before Before* are insignificant, and the coefficients before Post* are basically significant. This indicates that there is no significant difference in the corporate tax evasion (*ERT*) between the treatment group and the control group before the policy implementation, but there is a significant difference in the corporate tax evasion (*ERT*) between the treatment group and the control group after policy implementation. In other words, the parallel trend test of DID is satisfied. Meanwhile, from the coefficient before *Post*, it is evident that the impact of changing the social insurance collection institution from the social security department to the local tax department on corporate tax evasion is greatest in the second and third years after the policy is implemented, and in the fifth and sixth years after the policy is implemented, there is basically no policy effect.

**Table 3:** Parallel trend test-event analysis method.

Explained variable	*ERT*
Explanatory variables	(1)
*Before*4	0.006
	(1.513)
*Before*3	-0.004
	(-1.138)
*Before*2	0.002
	(1.362)
*Post*0	0.006***
	(3.301)
*Post*1	0.006***
	(3.359)
*Post*2	0.012***
	(5.920)
*Post*3	0.012***
	(5.905)
*Post*4	0.010***
	(4.618)
*Post*5	-0.001
	(-0.466)
*Post*6	0.004*
	(1.689)
Constant	0.456***
	(2.602)
Baseline controls	YES
Firm FE	YES
Year FE	YES
Observations	590911
Adj-R2	0.350

Note

The decomposition results in this table are calculated by stata17 and the script provided by Goodman-Bacon et al. (2021) [72]

#### 5.2.2. Bacon decomposition result

In Two-Way Fixed Effects Regression (TWFE), the TWFE estimator can be interpreted as the average causal effect of a policy only if the parallel trend assumption is satisfied between all groups and the causal effects of each group are not time-varying. In reality, policy effects are usually time-varying. In this case, even if the parallel trends assumption is satisfied, the TWFE estimator cannot be interpreted as an overall average causal effect. In order to estimate the impact of policy time-varying on the results of the fixed effects model, Goodman-Bacon proposed a method that can decompose the TWFE estimator into the weighted average of each part, which is also called Bacon decomposition. In this study, the reform of social insurance collection institutions is also time-varying. Therefore, we further verify the robustness of the baseline regression results through Bacon decomposition.

For the sake of robustness, we have carried out two rounds of Bacon decomposition. The first round considers all control variables but only a preliminary decomposition can be carried out. This result is shown in Table 4 Column (1) and (2). As we can see in Table 4 Column (2), the impact of changing the social insurance collection institution from the social security department to the local tax department on corporate tax evasion mainly comes from the difference between the whole treatment group and the control group (the weight is 0.924). Moreover, this effect is positive, which is consistent with the result in the baseline regression. Table 4 Column (3) and (4) presents the result of Bacon decomposition did not include control variables. This result also shows that the effect between the pilot city sets with different policy launch times accounted for a negligible weight. Most of the effect of changing the social insurance collection institution from the social security department to the local tax department on corporate tax evasion comes from the difference between the whole treatment group and the control group (cities never be treated, the weight is 0.954). This time, the effect is also positive, which is consistent with the result in the baseline regression.

**Table 4:** Bacon decomposition results.

Decomposition considering control variables	Beta	Weight	Decomposition without considering control variables	Avg. Diff.-in-diff. Estimate	Weight
	(1)	(2)		(3)	(4)
Treated groups	-0.009	0.001	Earlier treated groups vs. Later treated groups	0.034	0.041
Treated groups vs. Never treated groups	0.065	0.924	Later treated groups vs. Earlier treated groups	-0.02	0.005
Within	0.010	0.075	Treated groups vs. Never treated group	0.062	0.954

Note

(1) The T-statistic after clustering is in parentheses; (2) ***, **, and * indicate significance at the 1%, 5%, and 10% levels, respectively.

Therefore, although we use staggered DID in the baseline regression, which may lead to the problem of heterogeneity treatment effects. After bacon decomposition, we found that this heterogeneity effect problem was less influential. Therefore, the baseline regression results in this paper are credible.

#### 5.2.3. Excluding the impact of endogeneity

In order to exclude the impact of endogeneity, we conduct PSM-DID, Heckman two-step method, and placebo test in this section. We adopt the propensity score matching (PSM) method to select the enterprises in the control group to exclude the non-randomness in the selection of the treatment group and the control group. We selected the treatment and control groups using 1:1 nearest-neighbor matching without replacement. Table 5 Column (1) present the result of PSM-DID. The core explanatory variable *So*_*In* and actual tax rate *ERT* still have a significant positive relationship at the 1% statistical level. Therefore, the benchmark results of this study are robust, that is, the positive relationship between changing the social insurance collection institution and corporate tax evasion is less affected by the sample selection problem.

**Table 5:** Robustness Test 1.

Explained variable	*ETR*			*Vat*
	PSM-DID	Heckman two-step method	Virtual year placebo	VAT burden
Explanatory variables				
	(1)	(2)	(3)	(4)
*Mi*		0.008*		
		(1.829)		
*So*_*In*	0.007***	0.007***	0.004	0.305
	(3.120)	(4.657)	(1.630)	(0.907)
Baseline controls	YES	YES	YES	YES
Firm FE	YES	YES	YES	YES
Year FE	YES	YES	YES	YES
Observations	133807	590911	590911	590911
Adj-R2	0.347	0.350	0.350	0.237

Note

(1) The T-statistic after clustering is in parentheses; (2) ***, **, and * indicate significance at the 1%, 5%, and 10% levels, respectively.

The PSM-DID alleviates some sample self-selection problems, but there may still have some human factors in matching, such as which matching method and matching distance to choose that may affect the results. In addition, using PSM-DID will result in the loss of a large part of the samples, resulting in inaccurate estimation results. Therefore, we used the Heckman two-step method to further solve the problem of sample self-selection. First, the Probit model is used to estimate the factors affecting changing the social insurance collection institution, and the inverse Mills ratio (*Mi*) is calculated thus. Thereafter, we introduce *Mi* into Model (1) as a control variable to control the selection bias caused by changing the collection institution. The empirical results are shown in Column (2) of Table 5. The inverse Mills ratio *Mi* is significant at the 10% statistical level, indicating that there is indeed a sample self-selection problem in the model. However, after controlling for the sample self-selection problem, the core explanatory variable *So_In* and actual tax rate *ERT* still have a significant positive relationship at the 1% statistical level. This indicates that the results of this study are less affected by the sample self-selection problem.

Furthermore, we conducted a placebo test by constructing a false change in the timing of the social insurance collection institution. Specifically, we take two years before the actual changing of the social insurance collection institution as the time for the change, construct the dummy variable of the false period, and then estimate it according to the benchmark Model (1). The empirical results are shown in Column (3) of Table 5. At this time, the core explanatory variable *So_In* has no significant correlation with the actual tax rate *ERT*. This indicates that the decline in corporate tax evasion is indeed caused by changing the social insurance collection institution, rather than the impact of other events in random years.

For a long time, China’s value-added tax (VAT) collection has adopted the method of “controlling tax by ticket.” Therefore, it is more convenient for tax institutions to obtain value-added tax information, and the collection of this tax is relatively transparent and standardized. However, China’s corporate income tax usually adopts the method of “account audit and collection,” wherein there is more room for corporate tax evasion. Therefore, in theory, the impact of changing the social insurance collection institution from the social security department to the local tax department on corporate tax evasion should be more evident in the income tax category, while the impact on enterprises’ VAT collection may be unobvious. We refer to the method of Ali et al. (2015) [73] and Fan and Peng (2017) [74] and divide the VAT paid by the enterprise in the current period by the pre-tax accounting profit to construct the VAT tax indicator. We add it as an explained variable to benchmark Model (1) and re-regress it thereafter. The empirical results are shown in Column (4) of Table 5. The coefficient of *So_In* is not significant at this time. This indicates that changing the social insurance collection institution from the social security department to the local tax department does not affect the VAT burden of enterprises.

#### 5.2.4. Excluding the impact of omitted variables in the sample

In this section, we conduct a series of test to alleviate the influence of omitted variables and extreme values in the sample. To alleviate the problem of omitted variables, we continue to control for other variables at the provincial level based on Model (1), such as the proportion of the primary industry, the proportion of the secondary industry, the proportion of fiscal revenue and fiscal expenditure, the pressure of social security payments, the proportion of exports and GDP, and the proportion of interest payments and profits at the enterprise level. The empirical results are shown in Column (1) of Table 6. Moreover, we continue to relax the assumptions of Model (1), allowing enterprises to have different linear trends in tax evasion across provinces. Therefore, we control for the trend term of provinces based on the baseline Model (1). The empirical results are shown in Column (2) of Table 6. As shown in Column (1) and (2) in Table 6, the core explanatory variable *So_In* and actual tax rate *ERT* still have a significant positive relationship at the 1% statistical level. This indicates that, after controlling for more variables to alleviate the problem of missing variables, changing the social insurance collection institution from the social security department to the local tax department still significantly reduces corporate tax evasion.

**Table 6:** Robustness Test 2.

Explanatory variables	Control more variables	Control province trend	Exclude extreme values
	(1)	(2)	(3)
*So*_*In*	0.008***	0.007***	0.007***
	(5.310)	(5.034)	(4.773)
Baseline Controls	YES	YES	YES
Firm FE	YES	YES	YES
Year FE	YES	YES	YES
Observations	589637	590911	590911
Adj-R2	0.350	0.350	0.362

Note

(1) The T-statistic after clustering is in parentheses; (2) ***, **, and * indicate significance at the 1%, 5%, and 10% levels, respectively.

To reduce the influence of extreme values in the sample data on the estimation results, we perform an up and down tail reduction of 1% for all continuous indicator variables and re-regress Model (1) in this section. The empirical results are shown in Column (4) of Table 6. The core explanatory variable *So_In* and actual tax rate *ERT* still have a significant positive relationship at the 1% statistical level. This indicates that, after considering the impact of extreme values on the estimated results, changing the social insurance collection institution from the social security department to the local tax department still significantly deters corporate tax evasion.

#### 5.2.5. Excluding the impact of various events and policy during the sample periods

During the sample period, China have experienced different events shock and conducted various policy that may influence the baseline results. In 2001, China accessed to the World Trade Organization (WTO). This may leds to a decrease in the tariff rate on intermediate products of Chinese enterprises and then affect the effective income tax rate. To eliminate the interference of China’s entry into the WTO, we continue to control the ratio of enterprises’ exports to total output based on Model (1) in this section. The result is presented in Table 7 Column (1). In 2002, China eased its controls on foreign investment. Previous literature has shown that foreign capital liberalization may deter the improvement of corporate productivity [75], and corporate productivity will affect corporate profits, eventually leading to corporate tax evasion. The liberalization of foreign capital is mainly based on changes at the industry level, and the impact on the industry level varies according to the proportion of foreign investment in enterprises. Therefore, we construct an index of foreign capital entry intensity at the industry level in this section to alleviate the impact of foreign capital liberalization on the estimated results. The result is presented in Table 7 Column (2). China abolished the agricultural tax in 2005. The abolition of the agricultural tax increases the financial pressure of local governments, which may in turn force local governments to strengthen taxation efforts on enterprises, and finally increase the tax burden on enterprises. To exclude the influence of the 2005 agricultural tax, we restrict the sample to the 1998–2004 period and re-regress Model (1) in this section. The result is presented in Table 7 Column (3). In 2002, China implemented an income tax reform. The reform requires that the tax collection institution of newly established enterprises be unified into the state tax institution. Research shows that such changes in tax collection institution lead to corporate tax evasion. Therefore, the 2002 income tax reform may have led to biased estimates in this study and affected the research conclusion. To exclude the interference of the income tax reform in 2002, we removed the sample of enterprises established after 2002 and re-regressed Model (1). The result is presented in Table 7 Column (4). Furthermore, during the study’s sample period, China issued several fiscal and tax policies, such as the one for the software industry (Guo Shui Fa [2000] No. 24) or the energy infrastructure industry ([1999] No. 132). To control for the impact of such industry-oriented fiscal and taxation policies on the actual tax rate of enterprises, we refer to Liu and Zhao (2019) [34] and control the interaction term of industry and time based on Model (1). The result is presented in Table 7 Column (5).

**Table 7:** Robustness Test 3.

Explanatory variables	Control the WTO event	Control free foreign investment	Exclude agricultural tax	Exclude income tax	Exclude other policy effect
	(1)	(2)	(3)	(4)	(5)
*So*_*In*	0.006***	0.007***	0.005***	0.007***	0.007***
	(4.040)	(5.049)	(3.441)	(4.843)	(4.895)
Baseline controls	YES	YES	YES	YES	YES
Firm FE	YES	YES	YES	YES	YES
Year FE	YES	YES	YES	YES	YES
Observations	476677	590911	339034	511577	590911
Adj-R2	0.353	0.350	0.372	0.357	0.351

Note

(1) The T-statistic after clustering is in parentheses; (2) ***, **, and * indicate significance at the 1%, 5%, and 10% levels, respectively.

As we can see in Table 7, all the coefficients of *So_In* is significantly positive at the 1% statistical level. This indicates that, after considering the impact of various events and policy shocks, changing the social insurance collection institution from the social security department to the local tax department still significantly deters corporate tax evasion.

### 5.3. Mechanism analysis

As discussed earlier, changing the social insurance collection institution from the social security department to the tax department can help the tax department obtain more information about the enterprise, thereby reducing information asymmetry between government and enterprise. Thus, for enterprises with high information asymmetry, changing the social insurance collection institution may further deters tax evasion. To test whether this mechanism exists, we draw on Ryan et al.’s (2014) definition of information asymmetry [76]. We believe that the higher the proportion of intangible assets in an enterprise, the more difficult it is for government to understand the information of the enterprise when evaluating it, and thus, will face more serious information asymmetry. We define the surrogate variable of information asymmetry as *Int_Asset*_*1*_, that is, the ratio of intangible assets to total assets. Meanwhile, given that most of the intangible assets of industrial enterprises are 0, we define the dummy variable *Int_Asset*_*2*_ as another indicator of information asymmetry. If the intangible asset of the enterprise is larger than 0, then *Int_Asset*_*2*_ = 1, otherwise *Int_Asset*_*2*_ = 0. Columns (1) and (2) in Table 8 show the empirical results. The coefficients of the core explanatory variables So_In*Int_Asset_*1*_ and So_In*Int_Asset_*2*_ are significantly positive. This result indicates that with an increase in information asymmetry, corporate tax evasion in the treatment group will decrease significantly after changing the social insurance collection institution. This provides empirical evidence that changing the social insurance collection institution from the social security department to the local tax department will deter corporate tax evasion through information asymmetry.

**Table 8:** Mechanism analysis.

Explained variable	*ERT*			
	Excludes 2003		Supplement 2003	
Explanatory variables	(1)	(2)	(3)	(4)
*So*_In*Int_*Asset*1	0.034**		0.020*	
	(2.422)		(1.668)	
*Int*_*Asset*1	0.010		-0.001***	
	(1.341)		(-14.442)	
*So*_In*Int_*Asset*2		0.004***		0.004***
		(2.710)		(2.697)
*Int*_*Asset*2		-0.000		-0.001
		(-0.281)		(-0.639)
*So*_*In*	0.007***	0.006***	0.007***	0.006***
	(4.837)	(4.114)	(4.714)	(3.868)
Baseline controls	YES	YES	YES	YES
Firm FE	YES	YES	YES	YES
Year FE	YES	YES	YES	YES
Observations	515303	515303	590911	590911
Adj-R2	0.354	0.354	0.350	0.350

Note

(1) The T-statistic after clustering is in parentheses; (2) ***, **, and * indicate significance at the 1%, 5%, and 10% levels, respectively.

As there is no intangible asset subject in the industrial enterprise database in 2003, we supplement the data in 2003 using the imputation method and then re-regress. Columns (3) and (4) in Table 8 show the empirical results. The coefficients of the core explanatory variables So_In*Int_Asset_*1*_ and So_In*Int_Asset_*2*_ are still significantly positive. Therefore, this section shows that changing the social insurance collection institution from the social security department to the tax department reduces the information asymmetry between the government and the enterprise, while it deters corporate tax evasion.

### 5.4. Heterogeneous analysis

#### 5.4.1. Distinguishing corporate tax collection institution

The income tax reform implemented in China in 2002 used a “one size fits all” approach to the scope of tax collection and administration, which stipulated that non-state-owned enterprises/non-foreign-funded enterprises that were in the local tax bureau before the reform would remain in the local tax bureau, while all newly established enterprises since 2002 would pay corporate income tax at the state tax department. The division of tax jurisdiction makes changing the social insurance collection institution implemented in various provinces have different impacts on different enterprises. In theory, changing the social insurance collection institution to the local tax department will not affect the local tax department to verify the information of enterprises whose income tax is collected by the state tax department. After the change, the tax information of these enterprises is in the state tax department and the social security information is in the local tax department, while the local tax department still cannot realize the joint tax and social security audit of these enterprises. Enterprises whose income tax is levied by the local tax department will be more affected because the local tax department can retain more tax information and social security information of these enterprises. Therefore, we divide the sample into enterprises whose income tax is collected by the state tax department and those whose income tax is collected by the local tax department and study the impact of changing the social insurance collection institution on corporate tax evasion. Columns (1) and (2) in Table 9 show the empirical results. Enterprises whose income tax is collected by the state tax department is not affected by changing the social insurance collection institution, while enterprises whose income tax is collected by the local tax department are significantly affected.

**Table 9:** Heterogeneous analysis.

Explained variable	*ERT*					
	Local tax authority	Central tax authority	Labor intensive	Capital intensive	Low labor cost	High labor cost
Explanatory variables	(1)	(2)	(3)	(4)	(5)	(6)
*So*_*In*	0.007***	0.005	0.011***	0.002	0.008***	0.004*
	(4.753)	(0.433)	(5.138)	(1.012)	(3.916)	(1.941)
Baseline controls	YES	YES	YES	YES	YES	YES
Firm FE	YES	YES	YES	YES	YES	YES
Year FE	YES	YES	YES	YES	YES	YES
Observations	504808	79044	273637	273368	257011	259801
Adj-R2	0.356	0.325	0.353	0.366	0.320	0.398

Note

(1) The T-statistic after clustering is in parentheses; (2) ***, **, and * indicate significance at the 1%, 5%, and 10% levels, respectively.

#### 5.4.2. Distinguishing the factor-intensive types of enterprises

In this section, we analyze whether changing the social insurance collection institution, which has different effects on the enterprise, is labor- or capital-intensive. Lu et al. (2017) report that the higher the labor intensity of the enterprise, the greater its dependence on labor, and the more difficult it is for the enterprise to reduce its employees and their wages [77]. Changing the social insurance collection institution mainly reflects that the local tax department has obtained more social insurance information about workers. If a company is a labor-intensive enterprise, its social insurance information is relatively large. Therefore, the local tax department will receive more social insurance information and the supervision of the enterprise will be stronger, thereby deterring corporate tax evasion. We use the ratio of net fixed assets to the number of employees to determine whether a firm is labor- or capital-intensive. If the ratio of net fixed assets to employees is less than the sample median, it is a labor-intensive enterprise, and if it is greater than that, it is a capital-intensive enterprise. Columns (3) and (4) in Table 9 show the empirical results. In labor-intensive enterprise samples, the core explanatory variable *So_In* has a significant positive relationship with the explained variable *ERT*, but this relationship does not exist in the sample of capital-intensive enterprises. This indicates that after the social security collection institution has changed from the social security department to the local tax department, enterprises with greater dependence on labor are subject to stronger external supervision, resulting in less tax evasion.

#### 5.4.3. Distinguishing enterprise labor costs

In this section, we analyze whether the impact of changing the social insurance collection institution on corporate tax evasion varies in companies with different salary structures. In theory, enterprises can avoid tax by offering their employees the minimum wage and then repaying them in the form of performance or year-end bonuses. Therefore, corporate tax evasion is higher for enterprises with lower labor costs. However, when the social insurance collection institution was changed to the local tax department, the information asymmetry between the local tax department and the enterprise was reduced. The local tax department can strengthen the external governance of enterprises, thereby leading to a decrease in corporate tax evasion. Therefore, we can expect that, in the sample of low labor cost enterprises, changing the social insurance collection institution has a greater impact on corporate tax evasion. We use the company’s compensation expenditure to represent its compensation structure (deflated by total assets). If the ratio of enterprise salary expenditure to assets is greater than the median, the enterprise is a high labor cost enterprise; otherwise, it is a low labor cost enterprise. Columns (6) and (7) in Table 9 show the empirical results. In the sample of low labor cost enterprises, the core explanatory variable *So_In* has a significant positive relationship with the explained variable *ERT*, but there is no such relationship between the two in the sample of high labor cost enterprises. Therefore, it also indicates that after the social insurance collection institution changes from the social security department to the local tax department, enterprises that have previously relied on low labor costs will be subject to stronger external supervision, thus resulting in a decline in tax evasion.

## 6. Conclusions and policy implications

### 6.1. Conclusions

Exploiting the perfect quasi-natural experiment of the social insurance collection institution reform implemented in China in 2000, we examine the impact of changing the social insurance collection institution on micro-enterprise tax evasion from an information asymmetry perspective. The research results show that changing the social insurance collection institution from the social security department to the local tax department will significantly deter corporate tax evasion. This conclusion is also supported by a series of robustness tests, such as the parallel trend test, Heckman two-step method, PSM-DID, Bacon decomposition and placebo test. Since the information asymmetry between the government and enterprises is an important reason for corporate tax evasion [8, 10], in the mechanism analysis, we verified that changing the social insurance collection institution reduced the information asymmetry between the social insurance collection institution and the enterprise, thereby strengthening the external governance of the enterprise and deterring tax evasion. Furthermore, we find that the impact of changing the social insurance collection institution on corporate tax evasion is more evident in the samples of local tax enterprises, labor-intensive enterprises, and low labor cost enterprises.

### 6.2. Policy implications

This study has certain policy implications for the current institutional reform in China and for developing countries, especially the reform of social insurance collection institutions. In March 2018, the Chinese central government issued the “Plan for Deepening the Reform of Party and State Institutions,” proposing that various social insurances, such as endowment insurance and medical insurance, should be collectively handed over to the tax authorities for collection. By studying the implementation of the reform of the social security collection system in 2000, we demonstrate that the reform of the social security collection system will increase the tax department’s information vis-à-vis the enterprise, which will help the tax department to form a stronger external governance constraint on the enterprise and restrain it from tax evasion. This conclusion supports the positive economic benefits of the institutional reforms proposed by the Chinese central government. Meanwhile, this conclusion is also of great significance for the big data platform and information infrastructure established by the country to assist government governance. As we all know, there are high costs involved in institutional reforms and tax enforcement. This study proves that the role of social security collection institution reform is to reduce the information asymmetry between the government and enterprises, which is conducive to the government’s stricter supervision of corporate tax evasion, thereby deterring corporate tax evasion. With the development of information technology, there is increasing evidence prove that a more effective use of information and IT can be beneficial for revenue administrations in low-income countries [1, 78]. therefore, by strengthening data collection and establishing more advanced information technology processing equipment, the government can strengthen the investigation of information, such that its affairs can be managed more effectively.

### 6.3. Limitation and future research

Although this study contributes to the existing knowledge base, it has some limitations. First, we mainly use the ratio of the income tax payable to pre-tax accounting profit as a proxy indicator when calculating the indicator of corporate tax evasion. Therefore, this only indicate that changing the social insurance collection institution from the social security department to the tax department will affect the corporate tax avoidance behavior of income tax. Mascagni (2018) find that existing research about corporate tax avoidance mainly focuses on income tax, and there is not much discussion on other taxes such as value-added tax [20]. Future research can expand on this research question from the perspective of different tax types. Second, we only examine the short-term causal effect of government institution settings on corporate tax evasion. Much of the existing literature about the determinants of corporate tax avoidance only discusses its short-term effects on corporate tax evasion. However, its effect may not be sustainable over time [79]. Future research could further examine the long-term effects of government institution settings on corporate tax evasion.
